# Supplementary material for: Increased bacterial taxonomic and functional diversity is associated with impaired rotavirus vaccine immunogenicity in infants from India and Malawi
Source: BMC Microbiol. 2023 Nov 18;23:354. doi: 10.1186/s12866-023-03098-z (PMC10656894; doi:10.1186/s12866-023-03098-z)
Supplement: Supplementary file 6 — Additional file 6: Supplementary Figure 1. Summary of study design. Supplementary Figure 2. Comparison of 16S and metagenomic sequencing profiles. Supplementary Figure 3. Association between microbiome composition and rotavirus-specific IgA. Supplementary Figure 4. Association between microbiome composition and dose 1 oral rotavirus vaccine shedding. Supplementary Figure 5. Volcano plots of geometric mean rotavirus-specific IgA ratios in relation to feature presence or absence. Supplementary Figure 6. Volcano plots of feature prevalence in relation to dose 1 rotavirus vaccine shedding. [file 12866_2023_3098_MOESM6_ESM.pdf]

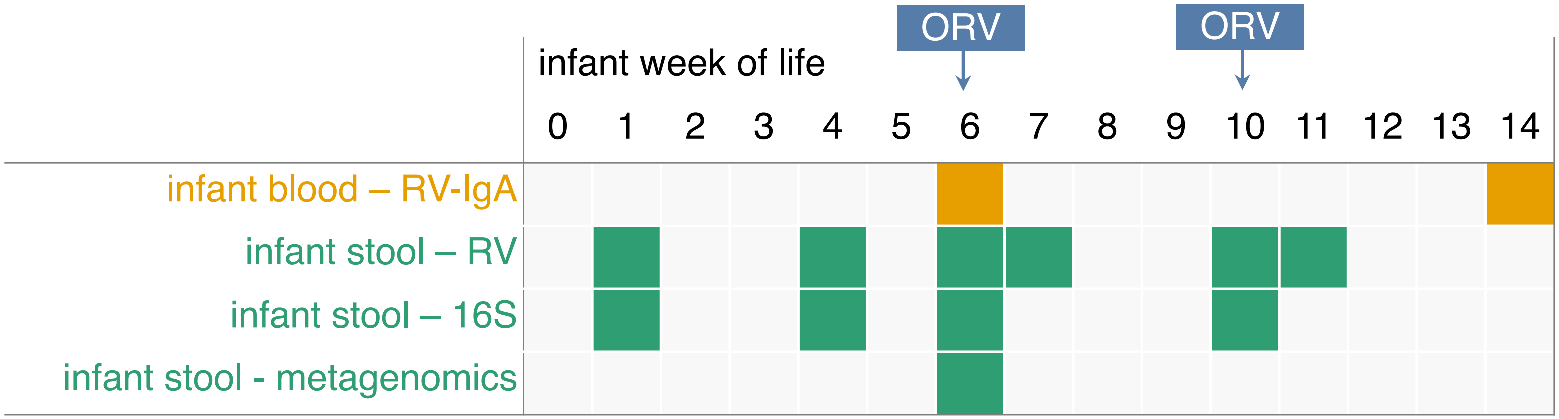

Supplementary Figure 1: Summary of study design. The study included infants from India (n = 283) and Malawi (58). It is nested within a larger cohort study that also included infants in the United Kingdom (n = 60). The full cohort also included additional sample types and assays including maternal RV-IgA, cord blood RV-IgA, breastmilk RV-IgA, breastmilk 16S, and biomarkers of environmental enteric dysfunction. ORV, oral rotavirus vaccine; RV, rotavirus.

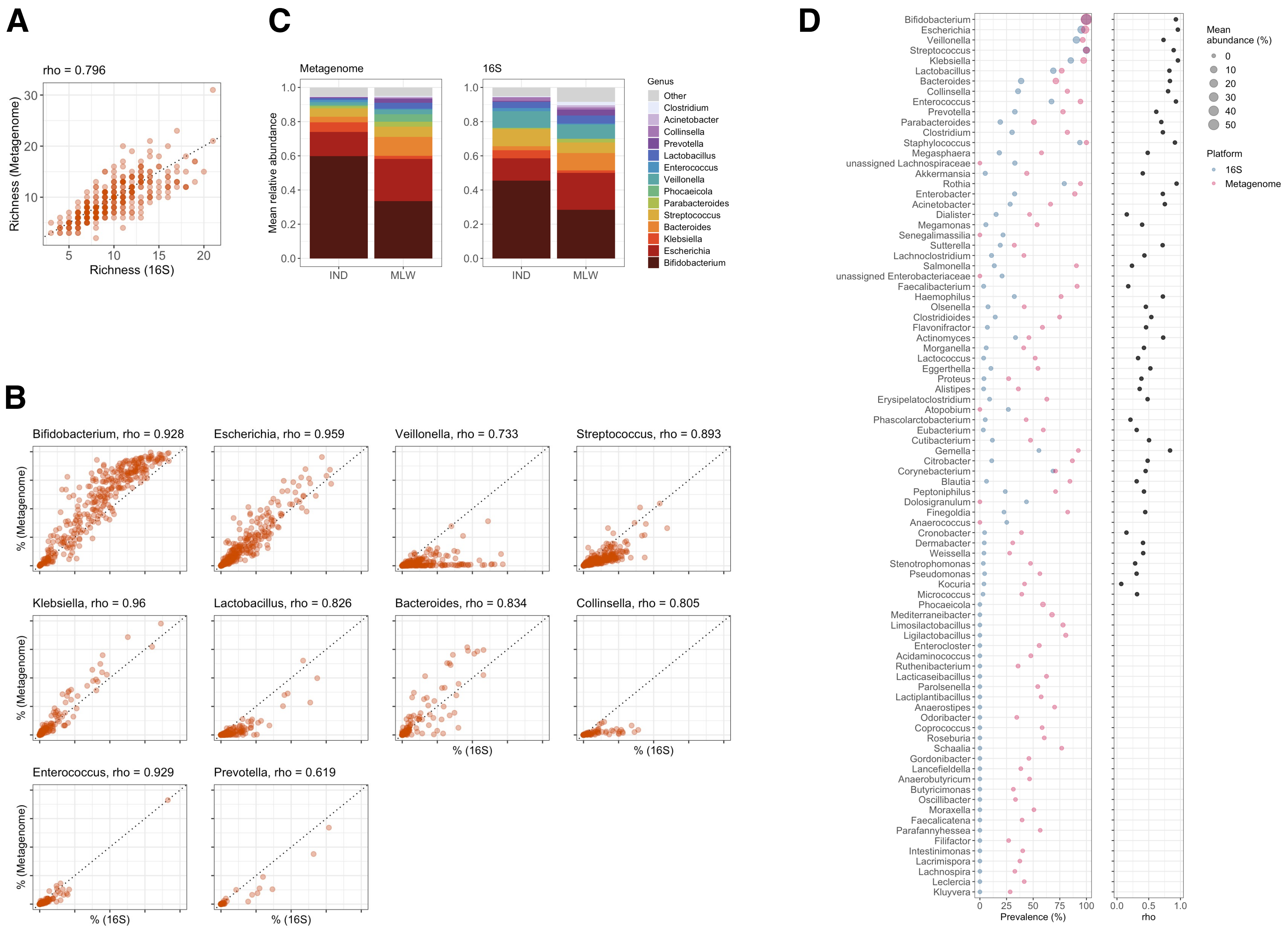

Supplementary Figure 2: Comparison of 16S and metagenomic sequencing profiles. (A) Comparison of genus-level richness estimates. Correlation was assessed via Spearman's rank correlation coefficient (rho). (B) Comparison of genus relative abundances for 10 most common genera in 16S data. (C) Mean genus abundances by country. Genera with a mean abundance of at least 1% in either country based on either metagenomic or 16S profile are displayed. (D) Prevalence, abundance, and correlation profiles for genera present in at least 20% of samples based on either metagenomic or 16S profile. Correlation was assessed via Spearman's rank correlation coefficient (rho) for genera detected by both platforms.

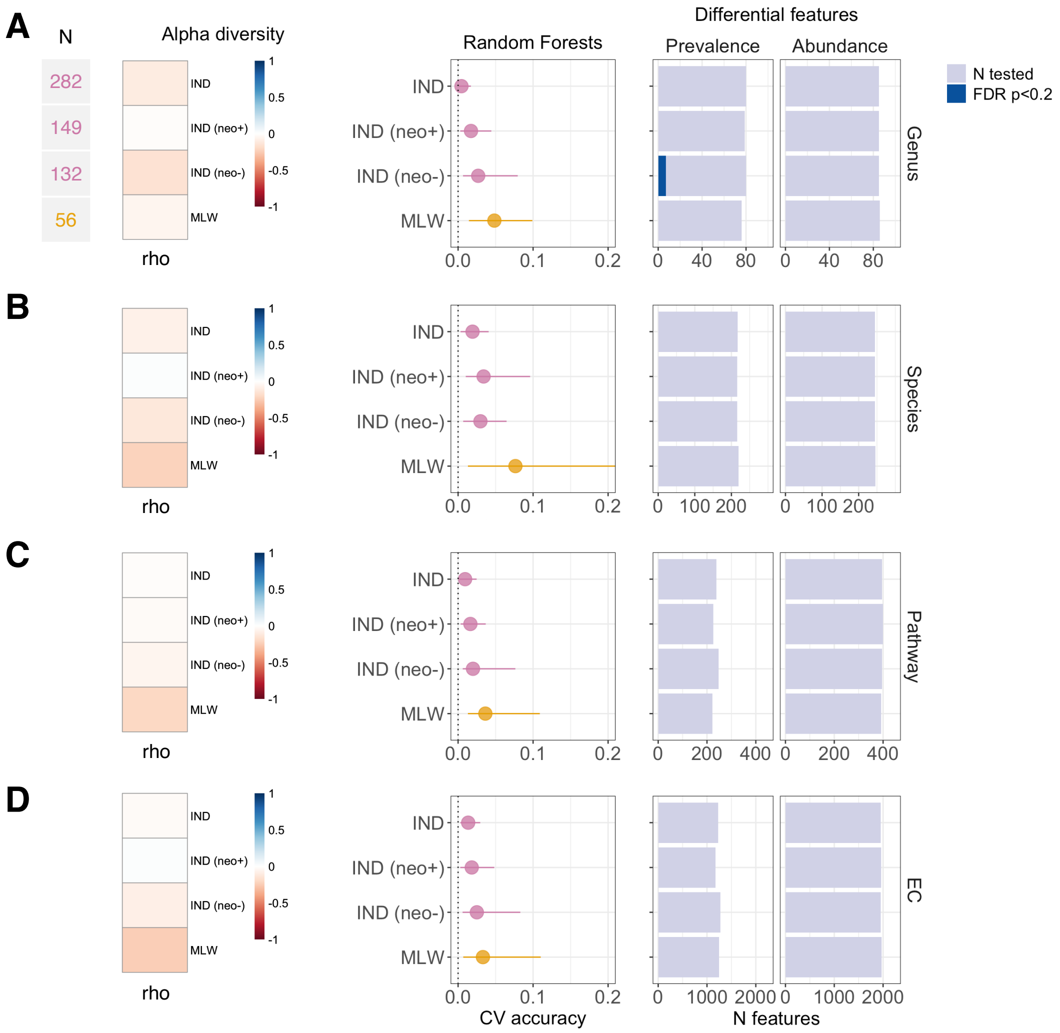

Supplementary Figure 3: Association between microbiome composition and rotavirus-specific IgA. Data are shown for (A) genus, (B), species, (C) pathway, and (D) enzyme commission data. The left panels display correlations between feature richness and post-vaccination RV-IgA concentration, as determined via Spearman's rank correlation coefficient (rho) with two-sided hypothesis testing. The middle panels display the cross-validation accuracy of Random Forests for prediction of post-vaccination RV-IgA. Median out-of-bag R^2 and interquartile range are displayed for predicted vs observed RV-IgA across 20 iterations of 5-fold cross-validation. The right panels display the number of enriched features based on prevalence (Wilcoxon rank-sum test for RV-IgA concentration in individuals with versus without the feature in question) and abundance (MaAsLin2). Analyses for Indian infants are reported for the overall cohort and stratified by neonatal wild-type rotavirus exposure. CV, cross-validation; EC, enzyme commission; FDR, false discovery rate; IND, India; MLW, Malawi; n.s., not significant; neo+, infected with rotavirus neonatally (defined by detection of rotavirus shedding in week of life 1 or baseline seropositivity); neo-, uninfected with rotavirus neonatally.

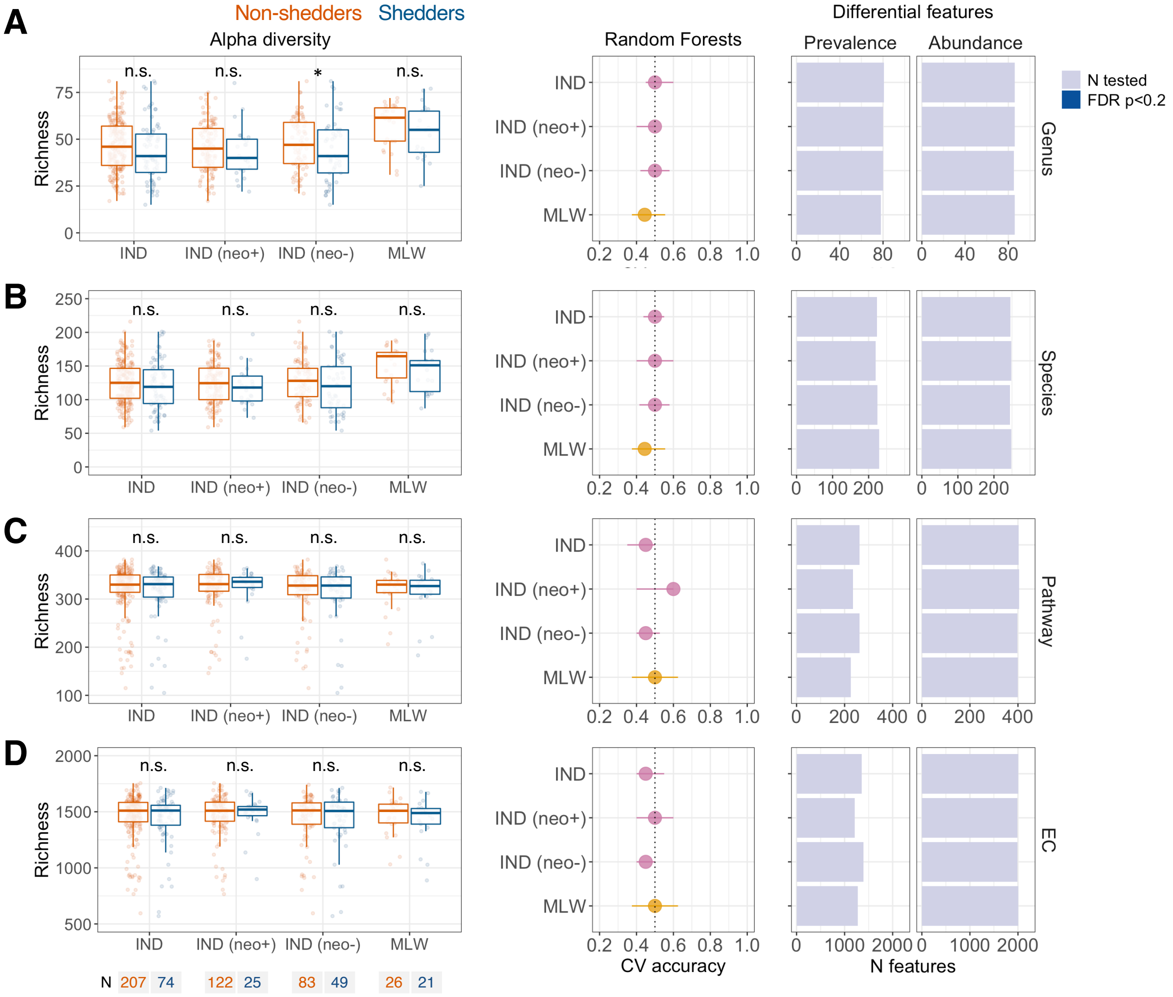

Supplementary Figure 4: Association between microbiome composition and dose 1 oral rotavirus vaccine shedding. Data are shown for (A) genus, (B), species, (C) pathway, and (D) enzyme commission data. The left panels display comparisons feature richness by shedding status. Groups were compared by Wilcoxon rank-sum test. The middle panels display the cross-validation accuracy of Random Forests for prediction of shedding status. Median out-of-bag accuracy (proportion correctly assigned) and interquartile range across 20 iterations of 5-fold cross-validation are displayed. Each iteration included an equal number of responders and non-responders (50 per group where possible, or else the number in the minority group if this was <50). The right panels display the number of enriched features based on prevalence (Fisher's exact test) and abundance (MaAsLin2). Analyses for Indian infants are reported for the overall cohort and stratified by neonatal wild-type rotavirus exposure. CV, cross-validation; EC, enzyme commission; FDR, false discovery rate; IND, India; MLW, Malawi; n.s., not significant; neo+, infected with rotavirus neonatally (defined by detection of rotavirus shedding in week of life 1 or baseline seropositivity); neo-, uninfected with rotavirus neonatally.

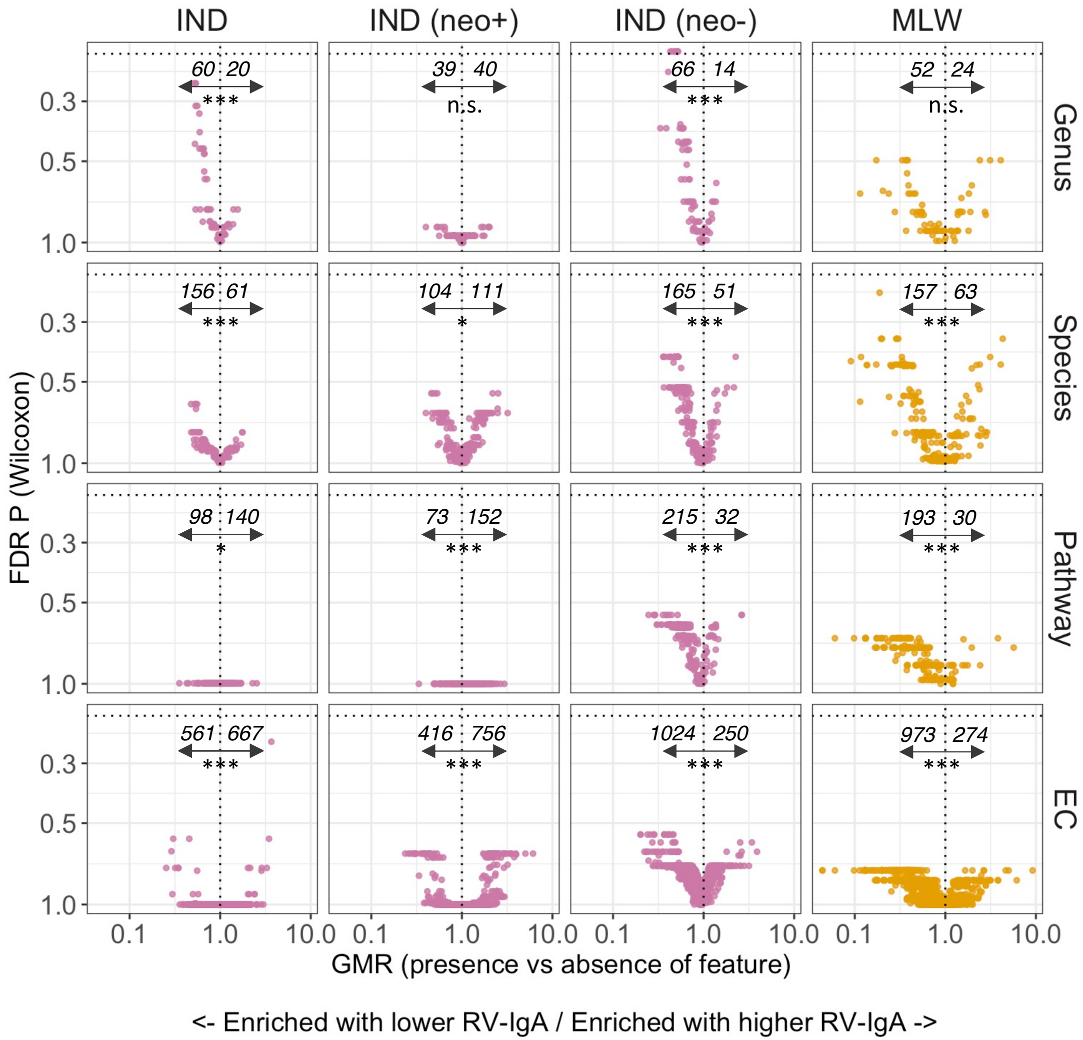

Supplementary Figure 5: Volcano plots of geometric mean rotavirus-specific IgA ratios in relation to feature presence or absence. Italicised numbers display the number of features with a prevalence difference of <1 (left of vertical dotted line) or ≥1 (right of vertical dotted line), highlighting features associated with lower and higher RV-IgA levels, respectively. The horizontal dotted line indicates an FDR p value of 0.2. The significance tests below the italicised numbers indicate the results of a Wilcoxon rank-sum test assessing skew in the distribution of associations (with a null hypothesis that negative and positive associations occur with equal frequency). Analyses for Indian infants are reported for the overall cohort and stratified by neonatal wild-type rotavirus exposure. EC, enzyme commission; FDR, false discovery rate; GMR, geometric mean ratio; IND, India; MLW, Malawi; n.s., not significant; neo+, infected with rotavirus neonatally (defined by detection of rotavirus shedding in week of life 1 or baseline seropositivity); neo-, uninfected with rotavirus neonatally; * p <0.05; *** p <0.0005.

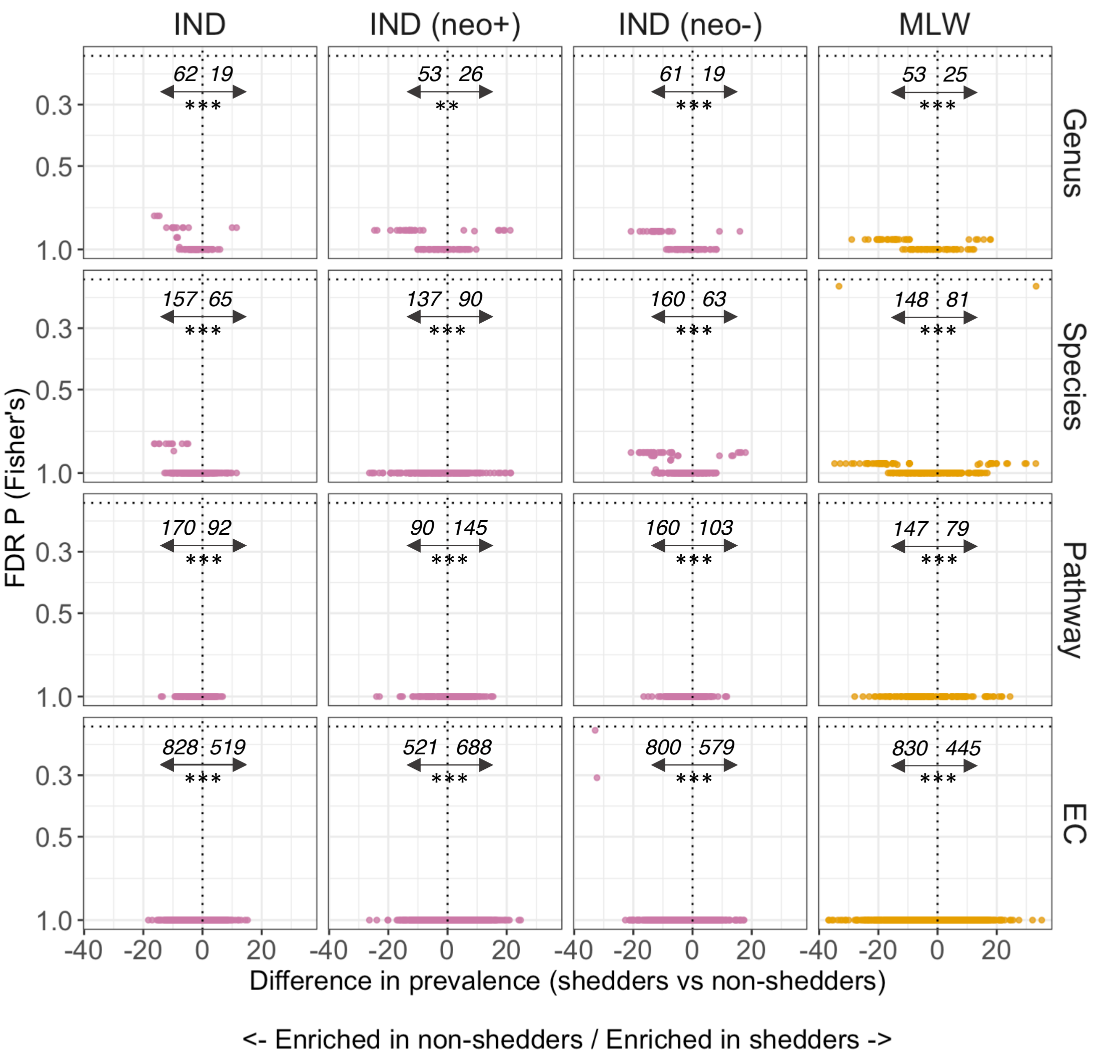

Supplementary Figure 6: Volcano plots of feature prevalence in relation to dose 1 rotavirus vaccine shedding. Italicised numbers display the number of features with a prevalence difference of <0 (left of vertical dotted line) or ≥0 (right of vertical dotted line), highlighting features enriched in shedders and shedders, respectively. The horizontal dotted line indicates an FDR p value of 0.2. The significance tests below the italicised numbers indicate the results of a Wilcoxon rank-sum test assessing skew in the distribution of associations (with a null hypothesis that negative and positive associations occur with equal frequency). Analyses for Indian infants are reported for the overall cohort and stratified by neonatal wild-type rotavirus exposure. EC, enzyme commission; FDR, false discovery rate; IND, India; MLW, Malawi; neo+, infected with rotavirus neonatally (defined by detection of rotavirus shedding in week of life 1 or baseline seropositivity); neo-, uninfected with rotavirus neonatally; sero+, seroconverters; sero-, non-seroconverters; ** p <0.005; *** p <0.0005.
